# Occurrence and antibiogram of *Listeria* species in raw pork, beef, and chicken meats marketed in Enugu State, Southeast Nigeria

**DOI:** 10.14202/vetworld.2020.317-325

**Published:** 2020-02-18

**Authors:** Onyinye Josephine Okorie-Kanu, Madubuike Umunna Anyanwu, Ekene Vivienne Ezenduka, Anthony Christian Mgbeahuruike, Christian Onwuchokwe Okorie-Kanu, Ejike Ekene Ugwuijem, Martha Nkechinyere Idogwu, Chidiebere Ohazuruike Anyaoha, Onyinye Lynda Majesty-Alukagberie, Roberto O. Vidal, Maricel Vidal

**Affiliations:** 1 Department of Veterinary Public Health and Preventive Medicine, University of Nigeria, 400001 Nsukka, Enugu State Nigeria; 2 Microbiology Unit, Department of Veterinary Pathology and Microbiology, University of Nigeria, 400001 Nsukka, Enugu State Nigeria; 3 Department of Veterinary Pathology, Michael Okpara University of Agriculture, 440233 Umudike, Abia State, Nigeria; 4 Department of Microbiology, University of Nigeria, 400001 Nsukka, Enugu State, Nigeria; 5 Programa de Microbiologia y Micologia, Instituto de Ciencias Biomedicas, Facultad de Medicina, Universidad de Chile, Santiago, Chile; 6 Secretaria Regional Ministerial de Salud Region Metropolitana Santiago, Chile

**Keywords:** antibacterial resistance, beef, chicken meat, *Listeria* species, pork

## Abstract

**Aim:**

This study was undertaken to isolate *Listeria* (*L*.) species from raw meats sold in markets in Enugu State, Southeast Nigeria, and to determine the antibacterial resistance profile.

**Materials and Methods:**

Twenty-five grams of beef (n=144), chicken meat (n=144), and pork (n=144) were collected randomly from supermarkets and general markets in Enugu State. Isolation of *Listeria* was done using half and full Fraser broths, and polymyxin acriflavine lithium chloride ceftazidime aesculin mannitol agar. Identification of isolates was done using an analytical profile index kit specific for *Listeria*. Confirmation of the genus *Listeria* was done by a polymerase chain reaction. The resistance of the isolates was determined using the disk diffusion method.

**Results:**

*Listeria* was isolated from 39/144 (27.1%) chicken meat, 19/144 (13.2%) pork, and 66/144 (45.8%) beef samples cultured. *Listeria* *innocua* was the predominant species in chicken meat (52.6%) and beef (81.8%) samples. *Listeria* *grayi*, *Listeria* *welshimeri*, and *Listeria* *ivanovii* were also isolated from the beef and chicken meat samples. More than 65% of the isolates were resistant to penicillin, rifampicin, ciprofloxacin, sulfamethoxazole-trimethoprim, and cephalothin. All the isolates from beef and pork samples and 23 (92%) from chicken meat samples, were resistant to ≥3 classes of antibacterial agents. Mean multiple antibiotic resistance index (MARI) was 0.77 (range=0.42-1.00), 0.58 (range=0.25-0.83), and 0.79 (range=0.58-0.92) for the isolates from beef, chicken meat, and pork samples, respectively. All the isolates had MARI >0.2.

**Conclusion:**

Multidrug-resistant *Listeria* strains contaminate raw beef, pork, and chicken meats marketed in Enugu State, Southeast Nigeria.

## Introduction

*Listeria* species are Gram-positive rods constituting part of normal commensal flora in the gut of humans and animals [1]. Due to their virulence attributes (biofilm formation, invasion and survival in host’s phagocytic cells, and so on), *Listeria* species are facultatively pathogenic and they are associated with zoonotic foodborne infections (generally termed listeriosis) worldwide [2]. The economic and health burden of listeriosis is outrageous. In the United States alone, the annual cost of foodborne listeriosis was estimated at US$2.3-22 billion while effective control of this disease potentially saves US$0.01-2 billion/year [3]. Reports of cases of *Listeria* infections concentrated in developed countries whereas cases in developing nations were unreported and underestimated [4]. However, the recent outbreak of listeriosis in South Africa (2017-2018) which accounted for 27% mortality (World Health Organization [WHO], 2018) [5], rekindled the interest in occurrence of *Listeria* species especially the antibacterial-resistant (ABR) strains, in foods of animal origin particularly in developing countries [2,5]. The WHO listed listeriosis as one of the notifiable diseases and issued a call for intensified surveillance of *Listeria* in foods of animal origin [5]. Because *Listeria* species are ubiquitous and highly adaptive tolerating adverse environmental conditions (such as low pH, high salinity and bile concentration, oxidative stress, carbon starvation, and so on), they easily contaminate slaughterhouse environment, especially where unhygienic slaughtering techniques are employed [6]. It is established that cross-contamination of meat and associated products at slaughterhouse, packaging and/or retailing stages, constitute putative risks for infection of individuals who make direct/indirect contact and/or consume these meat products [7]. Among the 18 currently known *Listeria* species [8], only *Listeria* *monocytogenes* and *Listeria* *ivanovii* were thought to be pathogenic being incriminated in several invasive diseases in humans and animals especially in immunocompromised individuals [6,9]. Today, other *Listeria* species such as *Listeria* *welshimeri* and *Listeria innocua* are increasingly isolated from diseased individuals [10,11].

Before now, infections caused by *Listeria* species were not difficult to treat because these organisms were considered susceptible to a wide variety of antibacterials [2,12]. But recently, the treatment of these infections has become increasingly difficult due to antibacterial resistance (particularly multidrug resistance [MDR]) [2,6]. Consequently, it is necessary to continually monitor the occurrence and antibacterial susceptibility of *Listeria* species from various settings [6]. Inappropriate use of antibacterials in humans and animals is the major cause of acquired resistance in *Listeria* species [2,6]. In Nigeria, the use of antimicrobials in the management of meat-yielding animals is uncontrolled; thus, veterinarians and non-professionals without veterinary supervision habitually use different types of antibacterial for prophylaxis and treatment of infections in these animals [13]. Thus, food animals (chicken, cattle, and pigs) slaughtered in Nigeria may be colonized by ABR *Listeria* spp. Meat derived from animals colonized by ABR *Listeria* can easily get contaminated with these organisms especially in slaughterhouses (as in Nigeria) where unhygienic slaughtering methods are employed and/or personal hygiene of the slaughterhouse workers are poor. Presence of ABR *Listeria* strains in meat is of public health importance because individuals who make direct or indirect contact with and/or consume these meats and associated products could acquire these organisms (and transfer them to their households/public) which are capable of transferring resistance genes by horizontal transfer to other bacteria in the gut of infected individuals. Compromise of subsequent antibacterial therapy in these individuals is a possible outcome and this has huge effects on public health.

In the available literature, studies from several countries [7,9,14-19] reported the occurrence and antimicrobial susceptibility of *Listeria* isolates from meats. In Nigeria, there are scanty reports in this regard, and these include studies in North-central [20,21], South-south [22-24], and South-west [4] regions of the country. The occurrence of ABR *Listeria* strains in foods of animal origin in Southeast Nigeria has remained uninvestigated. Moreover, identification of *Listeria* in the previous Nigerian studies was based on traditional biochemical tests which are not as reliable as genotypic methods. Thus, these studies might have underestimated or overrated the occurrence of *Listeria* in the meat samples.

This study aimed to isolate *Listeria* species from raw beef, pork, and chicken meats sold at markets in Enugu State Southeast Nigeria and to determine antimicrobial susceptibility profile of the isolates.

## Materials and Methods

### Ethical approval

Ethical approval was not necessary for this study. However, approval to conduct the study was obtained from the Department of Veterinary Public Health and Preventive Medicine, University of Nigeria.

### Study area

This study was conducted in Enugu State, Southeast Nigeria. The state comprised three senatorial/agricultural zones and 17 local government areas. Enugu State is located at latitudes 5°56’ North and 7°55’ North and longitudes 6°53’ East and 7°55’ East. Beef, pork, and chicken meats constitute the major animal protein consumed by the Enugu State population.

### Sampling

Between January 2018 and June 2018, three general markets (Ogige market in Nsukka, Abakpa, and Ogbete main markets in Enugu) and three supermarkets (all in Enugu metropolis) in Enugu State Southeast Nigeria, were visited 6 times each at weekly interval. In the general markets, potential meat buyers, as well as the butchers, frequently handle the meat while untrained personnel process and package meat for the supermarkets. A 25 g of chicken meat (n=144), beef (n=144), and pork (n=144) samples were collected and transported with ice packs to the laboratory of the Department of Veterinary Public Health and Preventive Medicine, University of Nigeria and processed on the day of collection.

### Isolation of Listeria species

This was done following the International Organization of Standardization’s 11290-1 guidelines [25]. Briefly, each sample was aseptically transferred into a sterile bag containing 225 mL pre-enrichment half-Fraser broth (HFB) (Oxoid, Basingstoke UK) with half-Fraser supplement, pulverized/homogenized using a stomacher for 1 min, and incubated at 30°C for 24 h in ambient air. Then, 0.1 ml of the HFB culture was inoculated into 10 ml enrichment full-Fraser broth (FFB) (Oxoid, Basingstoke UK) with full-Fraser supplement, and incubated at 37°C for 48 h in ambient air. A loopful of the FFB culture was inoculated on polymyxin acriflavine lithium chloride ceftazidime aesculin mannitol (PALCAM) agar (Oxoid, Basingstoke UK) with the supplement, and incubated at 37°C for 48 h. Suspected *Listeria* isolates (small-sized grayish-green colonies) were purified by sub-culturing/inoculating on fresh PALCAM agar with the supplement, and incubated at 37°C for 48 h. Pure cultures of the isolates were then inoculated on PALCAM agar slants with the supplement, incubated at 37°C for 48 h, and stored in a refrigerator at 4°C as stock cultures until needed for further analysis.

### Speciation of *Listeria* isolates

Phenotypic characterization of the isolates to species level was done by subjecting them to Gram staining and various biochemical tests such as catalase and hemolysis tests, as well as arylamidase (3,3′-diindolylmethane test), esculin hydrolysis, presence of α-mannosidase, and fermentation of D-arabitol, D-xylose, L-rhamnose, α-methyl-D-glucoside, D-ribose, glucose-1-phosphate, and D-tagatose using analytic profile index (API) *Listeria* kit (Biomerieux, France) as per the manufacturer’s instructions.

### Molecular confirmation/identification of the genus Listeria

Bacterial cultures were sub-cultured on PALCAM agar with supplement and incubated accordingly as above. Colonies of the isolates were inoculated into brain heart infusion broth (Oxoid, Basingstoke UK), and incubated at 37°C for 24 h. DNA of the isolates was extracted using bacterial DNA extraction and purification kit (Promega, USA) following the manufacturer’s directions. Using previously described protocols [18,26], DNA fragments of the *Listeria* specific *prs* gene of 370 bp were amplified by polymerase chain reaction (PCR) using prsF: 5’-GCTGAAGAGAGATTGCGAAAAGAAG-3’ and prsR: 5’-CAAAGAAACCTTGGATTTGCGG-3’ [26]. The PCR mixture was prepared in a total volume of 50 µl containing 5 of 10× buffer, 1.5 mM MgCl_2_, 250 µM each of the four dNTPs (Fermentes, Lithuania), 1.25 U of Taq DNA (Fermentes, Lithuania), 0.5 µM of each primer (IDT, USA), and 5 µL of template DNA [18]. The amplification was performed with an initial denaturation step at 94°C for 3 min; 35 cycles of 94°C for 0.40 min, 53°C for 1.15 min, and 72°C for 1.15 min; and one final cycle of 72°C for 7 min in a thermocycler (Eppendorf Mastercycler, Germany) [26]. Thereafter, 5 µl of the reaction mixture was mixed with 3 µl of loading buffer and separated on a 2% agarose gel in a TBE buffer (90 mM Trizma base, 90 mM boric acid, 2 mM EDTA, pH 8.3) [26]. The PCR product was visualized by ethidium bromide staining.

### Antibacterial susceptibility testing (AST)

Antibacterial susceptibility of 33, 25, and 10 *Listeria* isolates from beef, chicken meat, and pork samples, respectively, was determined by the disk diffusion method [27], using disks impregnated with 12 different antibacterial agents belonging to nine classes: β-lactam – penicillin (PEN, 10 units), amoxicillin (AMX, 10 µg), ampicillin (AMP, 2 µg), and cephalothin (CEF, 30 µg), nitroheterocyclics – nitrofurantoin (NIT, 300 µg), ansamycins – rifampicin (RIF, 5 µg), aminoglycosides – gentamicin (GEN, 10 µg), glycopeptides – vancomycin (VAN, 30 µg), tetracycline (30 µg), fluoroquinolones – ciprofloxacin (CIP, 5 µg), macrolides – erythromycin (ERY, 15 µg), and folate pathway inhibitors – sulfamethoxazole-trimethoprim (SXT, 25 µg). *L*. *monocytogenes* American Type Culture Collection (ATCC) 13932, *Streptococcus pneumoniae* ATCC 49619, and *Staphylococcus aureus* ATCC 29213, were used as reference strains. Results of the AST were interpreted according to the European Committee on Antimicrobial Susceptibility Testing [28] recommendations for *L*. *monocytogenes*, and Clinical and Laboratory Standards Institute [29] guidelines for *S. pneumoniae* (for the result of CEF) and staphylococci (for the results of PEN, AMX, NIT, RIF, VAN, TET, GEN, and CIP), respectively. Intermediately-susceptible isolates were classified as resistant. The multiple antibiotic resistance index (MARI) of the isolates was determined using the formula a/b where “a” is the number of antibacterial agents to which an isolate was resistant and “b” is the number of antibacterial agents to which an isolate was exposed [30]. Mean MARI was calculated as the ratio of total MARI and the total number of resistant isolates. An isolate resistant to ≥3 classes of antibiotics was considered multidrug-resistant [31].

### Statistical analysis

The frequencies of occurrence of *Listeria* spp. and resistance of the isolates were entered into Microsoft Excel version 2010 and their percentages and confidence intervals (CI) calculated.

## Results

### Occurrence of Listeria species in raw meat samples

*Listeria* was isolated from 39/144 (27.1%) chicken meat, 19/144 (13.2%) pork, and 66/144 (45.8%) beef samples cultured, respectively (Table-1). Of the 39 isolates from chicken meat, 31 (79.5%, 95% CI 66.8-92.2) were *L*. *innocua*, three (7.7%, 95% CI 0-16.1) each were *Listeria* *grayi* and *L*. *ivanovii* while 2 (5.1%, 95% CI 0-12.0) were *L*. *welshimeri*. The *L*. *welshimeri* and one *L*. *innocua* strain were recovered from the same sample. Among the 19 isolates from pork samples, nine (47.4%, 95% CI 31.7-63.1) were *L*. *innocua* whereas ten (52.6%, 95% CI 36.9-68.3) were *Listeria* species. Fifty-four (81.8%, 95% CI 69.7-93.9) of the 66 isolates from beef samples were *L*. *innocua* while four (6.1%, 95% CI 0-13.6) each were *L*. *grayi*, *L*. *welshimeri*, and *L*. *ivanovii*. Two of the *L*. *grayi* isolates and one *L*. *innocua* strain were obtained from two samples; all the *L*. *welshimeri* strains were isolated together with *L*. *innocua* in four samples whereas two *L*. *ivanovii* and two *L*. *grayi* strains were recovered from two different samples.

**Table-1:** Distribution of *Listeria* species in raw meats in Enugu State Southeast Nigeria.

Sample type	Number of samples cultured	Number (%) of isolates, 95% CI					Total (%)

		*L*. *innocua*	*L*. *ivanovii*	*L*. *grayi*	*L*. *welshimeri*	*Listeria* species	
Chicken meat	144	31 (79.5), 66.8-92.2	3 (7.7), 0-16.1	3 (7.7), 0-16.1	2 (5.1), 0-12.0	0 (0.0)	39 (27.1)
Pork	144	9 (47.4), 31.7-63.1	0 (0.0)	0 (0.0)	0 (0.0)	10 (52.6), 36.9-68.3	19 (13.2)
Beef	144	54 (81.8), 69.7-93.9	4 (6.1), 0-13.6	4 (6.1), 0-13.6	4 (6.1), 0-13.6	0 (0.0)	66 (45.8)

L=*Listeria*, CI=Confidence Interval

### Antibacterial susceptibility of Listeria isolates from raw meats

Among 33 isolates from beef, 31 (93.9%, 95% CI 85.9-100) were resistant to CEF, 30 (90.9%, 95% CI 87.7-94.1) to SXT and RIF, 29 (87.9%, 95% CI 76.8-99.0) to VAN and ERY, 26.9 (78.8%, 95% CI 64.9-92.7) to CIP, 22 (66.7%, 95% CI 39.1-94.3) to TET and GEN, 19 (57.6%, 95% CI 40.7-74.5) to AMP, 18 (54.5%, 95% CI 37.6-71.4) to NIT, and 16 (48.5%, 95% CI 31.4-65.6) to AMX (Table-2). Of the ten isolates from pork, all (95% CI 100) were resistant to CEF, CIP, and SXT, nine (90%, 95% CI 71.4-100) to RIF, eight (80%, 95% CI 55.2-100) to ERY, seven (70%, 95% CI 41.6-98.4) to NIT, VAN, and TET, six (60%, 95% CI 29.7-90.3) to GEN, and five (50%, 95% CI 19.1-80.9) to AMX and AMP. Out of 25 isolates from chicken meat, 24 (96%, 95% CI 88.4-100) were resistant to CEF, 18 (72%, 95% CI 54.4-89.6) to SXT and RIF, 15 (60%, 95% CI 40.8-79.2) to CIP, 14 (56%, 95% CI 36.5-56.0) to ERY, 11 (44%, 95% CI 24.5-63.5) to AMP, ten (40%, 95% CI 20.8-51.2) to VAN and TET, nine (36%, 95% CI 17.2-54.8) to GEN, six (24%, 95% CI 37.3-40.7) to NIT, and five (20%, 95% 4.3-35.7) to AMX. All the isolates were resistant to PEN. All the isolates from beef (33), pork (10), and 23 (92%) from chicken meat samples were resistant to ≥3 classes of antibacterial agents (Tables-3-5). Twenty-two, 21 and eight multiple ABR patterns (resistance to two or more antibacterial agents) were exhibited by isolates from beef, chicken meat, and pork, respectively. The resistance patterns PEN, CEF, AMX, NIT, VAN, TET, GEN, ERY, CIP, SXT, and RIF (n=4); PEN, CEF, VAN, GEN, CIP, AMP, SXT, PEN, CEF, TET, ERY, CIP, SXT, RIF, PEN, CEF, NIT, TET, ERY, CIP, SXT, RIF and PEN, CEF, AMX, NIT, VAN, GEN, ERY, CIP, SXT, and RIF (n=2 for each); and PEN, CEF, AMX, NIT, VAN, TET, GEN, ERY, CIP, SXT, and RIF (n=2) were predominant among isolates from beef, chicken meat, and pork, respectively. Mean MARI was 0.77 (range=0.42-1.00), 0.58 (range=0.25-0.83), and 0.79 (range=0.58-0.92) for beef, chicken meat, and pork isolates, respectively. All the isolates had MARI >0.2.

**Table-2:** Antibacterial susceptibility profile of *Listeria* isolates from raw meats marketed in Enugu State Southeast Nigeria.

Antibacterial class	Antibacterial agent (Concentration)	Number (%) of resistant isolates, 95% CI		

		Beef (n=33)	Chicken meat (n=25)	Pork (n=10)
β-lactam	Penicillin (10 units)	33 (100.0), 100	25 (100.0), 100	10 (100.0), 100
	Cephalothin (30 μg)	31 (93.9), 85.9-100	24 (96.0), 88.4-100	10 (100.0), 100
	Amoxicillin (10 μg)	16 (48.5), 31.4-65.6	5 (20.0), 4.3-35.7	5 (50.0), 19.1-80.9
	Ampicillin (2 μg)	19 (57.6), 40.7-74.5	11 (44.0), 24.5-63.5	5 (50.0), 19.1-80.9
Nitroheterocyclics	Nitrofurantoin (30 μg)	18 (54.5), 37.6-71.4	6 (24.0), 37.3-40.7	7 (70.0), 41.6-98.4
Glycopeptides	Vancomycin (30 μg)	29 (87.9), 76.8-99.0	10 (40.0), 20.8-51.2	7 (70.0), 41.6-98.4
Tetracyclines	Tetracycline (30 μg)	22 (66.7), 39.1-94.3	10 (40.0), 20.8-51.2	7 (70.0), 41.6-98.4
Aminoglycosides	Gentamicin (10 μg)	22 (66.7), 50.7-82.7	9 (36.0), 17.2-54.8	6 (60.0), 29.7-90.3
Macrolides	Gentamicin (10 μg)	29 (87.9), 76.8-99.0	14 (56.0), 36.5-56.0	8 (80.0), 55.2-100
Fluoroquinolones	Ciprofloxacin (5 μg)	26 (78.8), 64.9-92.7	15 (60.0), 40.8-79.2	10 (100.0), 100
Ansamycins	Rifampicin (5 μg)	30 (90.9), 87.7-94.1	18 (72.0), 54.4-89.6	9 (90.0), 71.4-100
Folate inhibitors	SUlphamethoxazole-trimethoprim (5 μg)	30 (90.9), 87.7-94.1	18 (72.0), 54.4-89.6	10 (100.0), 100

CI=Confidence Interval

**Table-3:** Multiple antibacterial resistance patterns and indices of 33 *Listeria* isolates from raw beef marketed in Enugu State Southeast Nigeria.

Number of antibacterial class	Resistance pattern (Number of isolates)	Total number of isolates (%)	MARI (Total)
3	PEN, CEF, VAN, AMP, RIF (1)	33 (100)	0.42 (0.42)
4	PEN, VAN, ERY, AMP, SXT (1)		0.42 (0.84)
	PEN, CEF, NIT, VAN, AMP (1)		
	PEN, CEF, AMX, VAN, CIP, AMP, RIF (1)		0.58 (0.58)
6	PEN, CEF, AMX, TET, ERY, CIP, SXT, RIF (1)		0.67 (0.67)
	PEN, CEF, NIT, VAN, GEN, SXT, RIF (1)		0.58 (0.58)
	PEN, VAN, TET, ERY, SXT, RIF (1)		0.50 (0.50)
	PEN, CEF, AMX, TET, GEN, ERY, CIP, AMP, SXT (1)		0.75 (0.75)
	PEN, CEF, VAN, TET, ERY, AMP, SXT, RIF (1)		0.67 (1.34)
	PEN, CEF, AMX, GEN, ERY, CIP, SXT, RIF (1)		
7	PEN, CEF, AMX, NIT, VAN, ERY, CIP, AMP, SXT, RIF (1)		0.83 (0.83)
	PEN, CEF, AMX, TET, GEN, ERY, CIP, SXT, RIF (1)		0.75 (1.50)
	PEN, CEF, VAN, TET, ERY, CIP, AMP, SXT, RIF (1)		
	PEN, CEF, VAN, GEN, ERY, CIP, SXT, RIF (1)		0.67 (0.67)
8	PEN, CEF, VAN, TET, GEN, ERY, CIP, AMP, SXT, RIF (3)		0.83 (6.64)
	PEN, CEF, NIT, VAN, TET, ERY, CIP, AMP, SXT, RIF (2)		
	PEN, CEF, AMX, NIT, VAN, TET, GEN, ERY, SXT, RIF (1)		
	PEN, CEF, AMX, NIT, VAN, GEN, ERY, CIP, SXT, RIF (2)		
	PEN, CEF, NIT, VAN, GEN, ERY, CIP, SXT, RIF (1)		0.75 (0.75)
9	PEN, CEF, NIT, VAN, TET, GEN, ERY, CIP, AMP, SXT, RIF (3)		0.92 (6.44)
	PEN, CEF, AMX, NIT, VAN, TET, GEN, ERY, CIP, SXT, RIF (4)		
	PEN, CEF, AMX, NIT, VAN, TET, GEN, ERY, CIP, AMP, SXT, RIF (3)		1.00 (3.00)

MARI=Multiple antibacterial resistance index, PEN=Penicillin, CEF=Cephalothin, AMX=Amoxicillin, NIT=Nitrofurantoin, VAN=Vancomycin, TET=Tetracycline, GEN=Gentamicin, ERY=Erythromycin, CIP=Ciprofloxacin, AMP=Ampicillin, SXT=Sulfamethoxazole-trimethoprim, RIF=Rifampicin

**Table-4:** Multiple antibacterial resistance patterns and indices of 25 *Listeria* isolates from raw chicken meats marketed in Enugu State Southeast Nigeria.

Number of antibacterial class	Resistance pattern (Number of isolates)	Total number of isolates (%)	MARI (Total)
1	PEN, CEF, AMP (1)	1 (4.0)	0.25 (0.25)
2	PEN, CEF, AMP, RIF (1)	1 (4.0)	0.33 (0.33)
3	PEN, CEF, AMP, SXT, RIF (1)	23 (92)	0.42 (1.68)
	PEN, CEF, VAN, CIP, AMP (1)		
4	PEN, VAN, TET, AMP, RIF (1)		
	PEN, CEF, VAN, AMP, SXT (1)		
	PEN, CEF, ERY, AMP, SXT, RIF (1)		0.50 (2.00)
	PEN, CEF, NIT, CIP, AMP, RIF (1)		
5	PEN, CEF, TET, ERY, SXT, RIF (1)		
	PEN, CEF, TET, CIP, SXT, RIF (1)		
	PEN, CEF, VAN, GEN, CIP, AMP, SXT (2)		0.58 (1.16)
6	PEN, CEF, AMX, VAN, GEN, ERY, SXT, RIF (1)		0.67 (0.67)
	PEN, CEF, TET, ERY, CIP, SXT, RIF (2)		0.58 (1.74)
	PEN, CEF, GEN, ERY, CIP, SXT, RIF (1)		
	PEN, CEF, AMX, TET, ERY, CIP, SXT, RIF (1)		0.67 (3.35)
7	PEN, CEF, NIT, TET, ERY, CIP, SXT, RIF (2)		
	PEN, CEF, NIT, TET, GEN, ERY, CIP, SXT (1)		
	PEN, CEF, VAN, GEN, ERY, CIP, SXT, RIF (1)		
	PEN, CEF, AMX, NIT, TET, ERY, CIP, AMP, SXT, RIF (1)		0.83 (0.83)
8	PEN, CEF, NIT, VAN, GEN, ERY, CIP, SXT, RIF (1)		0.75 (0.75)
	PEN, CEF, AMX, NIT, VAN, GEN, ERY, CIP, SXT, RIF (2)		0.83 (1.66)

MARI=Multiple antibiotic resistance index, PEN=Penicillin, CEF=Cephalothin, AMX=Amoxicillin, NIT=Nitrofurantoin, VAN=Vancomycin, TET=Tetracycline, GEN=Gentamicin, ERY=Erythromycin, CIP=Ciprofloxacin, AMP=Ampicillin, SXT=Sulfamethoxazole-trimethoprim, RIF=Rifampicin

**Table-5:** Multiple antibacterial resistance patterns and indices of 10 *Listeria* isolates from raw pork marketed in Enugu State Southeast Nigeria.

Number of antibacterial class	Resistance pattern (Number of isolates)	Total number of isolates (%)	MARI (Total)
5	PEN, CEF, AMX, ERY, CIP, SXT, RIF (1)	10 (100)	0.58 (0.58)
6	PEN, CEF, NIT, VAN, CIP, AMP, SXT, RIF (1)		0.67 (1.34)
	PEN, CEF, NIT, VAN, TET, CIP, AMP, SXT (1)		
7	PEN, CEF, NIT, TET, ERY, CIP, AMP, SXT, RIF (1)		0.75 (2.25)
	PEN, CEF, NIT, GEN, ERY, CIP, AMP, SXT, RIF (1)		
8	PEN, CEF, VAN, TET, GEN, ERY, CIP, SXT, RIF (1)		
	PEN, CEF, AMX, VAN, TET, GEN, ERY, CIP, AMP, SXT, RIF (1)		0.92 (3.68)
9	PEN, CEF, AMX, NIT, VAN, TET, GEN, ERY, CIP, SXT, RIF (3)		

MARI=Multiple antibiotic resistance index, PEN=Penicillin, CEF=Cephalothin, AMX=Amoxicillin, NIT=Nitrofurantoin, VAN=Vancomycin, TET=Tetracycline, GEN=Gentamicin, ERY=Erythromycin, CIP=Ciprofloxacin, AMP=Ampicillin, SXT=Sulfamethoxazole-trimethoprim, RIF=Rifampicin

## Discussion

In this study, *Listeria* was isolated from 27.1% chicken meat, 13.2% pork, and 45.8% beef samples suggesting that in Enugu State Nigeria, beef is more contaminated with the organisms than chicken meat and pork. This finding may be due to the larger surface area of cattle carcasses compared with meat carcass surface area of the other sampled animal types; therefore, the beef samples might have been contaminated more than the other meat-type samples. Possible sources of these organisms in the sampled meats include fecal contamination during slaughter/processing by endogenous *Listeria*, cross-contamination from slaughterhouse environment (as Nigerian slaughterhouses are usually in poor sanitary conditions), fomites (such as knives, wheelbarrow, meat-transport vehicles, meat display tables, and cloths used for cleaning tables), water used for washing carcasses and meat display tables, flies colonized with *Listeria*, and/or cloths/hands of butchers, meat-retailers, and/or buyers [15,23]. *Listeria* species are known to cross-contaminate meat samples from environmental contact surfaces due to their ability to attach to these surfaces using peritrichous flagella and to form biofilm [15].

The 27.1% occurrence in chicken meat in this study is higher than 6.67% and 21.6% occurrence of *Listeria* spp. in 60 each of randomly-selected raw and frozen chicken meat samples reported in Plateau State, North-central Nigeria, respectively [20]. However, it is lower than 40%, 47.5%, 44%, and 50% occurrence of *Listeria* in 200 fresh chicken carcasses reported in Iran [32], 160 fresh broiler chicken meat samples reported in Jordan [33], 25 frozen raw chicken fillets recorded in Egypt [15], and ten raw chicken meats observed in Saudi Arabia [19], respectively. In Korea, Arak, and Ardic [18] reported 51.66% occurrence of *Listeria* in 115 randomly-selected fresh turkey meat samples while 33.3% occurrence of *Listeria* was reported in 401 samples of poultry products in Iran [17].

The 45.8% occurrence in beef samples in this study is higher than 42.1% and 2.1% occurrence of *Listeria* in 50 randomly-selected raw beef samples reported in Rivers State, South-south Nigeria [23] and raw beef carcasses in India [34], respectively. However, it is lower than 52.78% occurrence of *Listeria* in 36 randomly-selected raw beef samples reported in Rivers State, South-south Nigeria [22]. It is also lower than 50% occurrence of *Listeria* in ten raw beef samples reported in Saudi Arabia [19]. A lower occurrence (13.2%) was recorded in raw pork samples in this study compared with 22% occurrence of *Listeria* in 50 pork samples reported by Odu and Okonko [23]. Other previous studies reported a 21.4-58.7% occurrence of *Listeria* in various types of meat samples [7,9,14,16,19]. The variation in the results is related to differences in the rate of meat carcass contamination, isolation, and identification methodologies. In the hereby experiment, the isolates were identified to genus level using PCR considered the “gold standard” for identification of *Listeria* which is difficult to be accurately identified by only phenotypic methods used in some of the previous studies [35]. Therefore, those studies, including the Nigerian reports, in which traditional phenotypic methods were solely used for the identification of *Listeria*, might have deemphasized or overestimated the occurrence of *Listeria* in the meat samples [18]. Nevertheless, similar to the findings of the previous studies on raw meat samples in Nigeria [20,22,23] and elsewhere [14-17,33,36], this study identified various *Listeria* species, including *L*. *ivanovii*, *L*. *innocua*, *L*. *welshimeri*, and *L*. *grayi* and unidentified *Listeria* species suggesting that diverse *Listeria* species contaminate retailed/marketed meats in Nigeria. The unidentified *Listeria* isolates in this study could belong to the remaining 14 of the currently known 18 *Listeria* spp. [1,8] or they may be species that are unidentifiable with the *Listeria* specific API kit employed in this experiment. Although *L*. *monocytogenes* (considered the most pathogenic *Listeria* species) was not identified in this work, the other identified species have been associated with invasive diseases (such as bacteremia) responsible for morbidity and mortality in humans and animals [10,11,37,38] and therefore calls for concern. It is noteworthy that in Rivers State South-south Nigeria, Odu and Okonko [23] reported 7% *L*. *monocytogenes* in 100 pooled raw beef/pork samples. Similarly, another study in Nigeria [4] focusing on *L*. *monocytogenes*, reported its detection in raw chicken meat samples. Therefore, *L*. *monocytogenes* is not an uncommon contaminant of raw meats in Nigeria and its occurrence should not be undermined.

In this study, *L*. *innocua* predominated among isolates from beef and chicken meat samples, while unidentified *Listeria* species dominated in the pork samples. This suggested that no one *Listeria* species could be regarded as a constant predominant contaminant of raw meats marketed in the study area. Odu and Okonko [23] also reported *L*. *innocua* as the dominant isolate whereas Eruteya *et al*. [22] and Daniel *et al*. [20] stated that *L*. *welshimeri* and *L*. *grayi* dominated in their respective studies, thus further indicating that no *Listeria* species constantly predominates in retailed raw meats in Nigeria. However, the dominance of *L*. *innocua* in beef and chicken meats in this study may suggest that this organism, as well as other *Listeria* species, are readily present in environments where these meats are processed. This calls for concern because *L*. *innocua* is genetically-related to *L*. *monocytogenes;* hence, they could easily exchange virulence/resistance genes [18]. Elsewhere, *L*. *innocua* [14] or *L*. *monocytogenes* [19,32] dominated among isolates from raw poultry meat samples. In raw beef samples, *L*. *monocytogenes* [36] or *L*. *welshimeri* [19] was the most commonly isolated.

The 81.8% occurrence of *L*. *innocua* in beef in this study is higher than 11-22.5% occurrence of *L*. *innocua* in raw beef samples reported by the previous studies [22,23,36]. The 79.5% occurrence of *L*. *innocua* in chicken meat samples in this study is higher than 28.5% and 28.7% occurrence of *L*. *innocua* in raw chicken meat samples reported by Daniel *et al*. [20] and Zenaili *et al*. [32], respectively. However, the occurrence of *L*. *innocua* in pork samples (47.4%) in this study is lower than 71.4% occurrence in raw pork samples reported by Odu and Okonko [22]. A 7.7% *L*. *grayi* and 6.1% *L*. *ivanovii* occurrences were recorded in chicken meat samples in this study whereas Abd El-Malik *et al*. [15] and Daniel *et al*. [20] reported a higher 16% and 12%, and 58.3% and 11.1% occurrences of these organisms in chicken meat samples, respectively. This study observed 6.1% occurrences each of *L*. *welshimeri*, *L*. *grayi*, and *L*. *ivanovii* in beef samples. Eruteya *et al*. [22] reported a higher 38.9% *L*. *grayi* and 97.2% *L*. *welshimeri* occurrences, and a lower 5.6% occurrence of *L*. *ivanovii* occurrence in beef samples. Odu and Okonko [23] recorded 50% (higher than the results of this study) occurrences of *L*. *grayi* and *L*. *ivanovii* in beef and pork samples, respectively. In Jordan, Al-Nabulsi *et al*. [36] reported a lower 2.9% *L*. *welshimeri*, and a higher 22.8% *L*. *ivanovii* and 8.6% *L*. *grayi* occurrences in 35 raw beef samples, respectively. The 5.1% occurrence of *L*. *welshimeri* in chicken meat samples in this study is similar to 5.6% occurrence of *L*. *welshimeri* in chicken meat samples reported by Daniel *et al*. [20], but it is lower than 8% occurrence of *L*. *welshimeri* in frozen raw chicken fillets reported by Abd El-Malek *et al*. [15].

The high resistance rates observed against PEN (100%), RIF (72-90.9%), CIP (60-100%), SXT (72-100%), and CEF (93.9-100%), in this study indicates selection pressure. Moderate to high percentages of the isolates in this study also preferred AMP (44-57.5%), AMX (20-50%), GEN (36-66.7%), TET (40-70%), ERY (56-87.9%), VAN (40-87.9%), and NIT (24-70%), further suggesting acquisition of genes encoding resistance to the agents following use-selection pressure. In Nigeria, the use of antibiotics in humans and meat-producing animals is uncontrolled and environmental sanitation is poor [13]; thus, these selection pressures might have emanated from diverse settings. The finding of high PEN and moderate to high AMP and GEN resistance in this study is worrisome because these drugs are first-line agents for treating listeriosis in humans and animals [6,39]. Thus, there may be limited options for therapy in individuals infected by isolates in this study. High rates of resistance against SXT, VAN, ERY, CIP, and RIF observed in this study is also disturbing because these drugs are combined and used as an alternative (second-line choice) for treating listeriosis, especially in pregnant women and patients that are allergic to β-lactams (PEN and AMP) [2,18,36,40,41]. Moreover, they are critically-important drugs of the highest priority for treating infections associated with Gram-positive bacteria [42]. Consumption of these resistant organisms together with the meats could result in the transfer of resistance genes by horizontal transfer to other bacteria flora in the gut of infected individuals. This could consequently jeopardize antibacterial therapy in these individuals who are potential reservoirs and disseminators of these organisms. The high rates of AMP, PEN, GEN, and TET resistance in this study are consistent with the result of the previous studies among *Listeria* isolates from poultry meats [14,15,17,43,44], but they contrasted Gowda and Van Damme [34] stating that most *Listeria* isolates from raw beef carcasses were susceptible to these drugs.

A high percentage (92-100%) of isolates in this study exhibited resistance to three or more classes of antibacterial agents tested, thus indicating that a high proportion of *Listeria* contaminating retailed raw meats in the study area is multidrug-resistant strains [31]. This finding is troubling because consumption of and/or contact with these contaminated meats could result in the acquisition of multidrug-resistant *Listeria* strains which are capable of transferring multiple resistance genes (encoding resistance to different antimicrobial classes) to bacteria flora in the gut of infected/colonized individuals. Consequently, these carriers could subsequently discharge these organisms (through feces) into the environment thereby posing risks to public health. In Nigeria, slaughterhouse workers, meat sellers/retailers, and potential meat-buyers (during negotiation especially in general markets) frequently handle processed/displayed raw meats. In addition, synanthropic flies often perch on these meats (which are often displayed in the open air) and thus could potentially transfer multidrug-resistant *Listeria* to other ecological niches. These are putative risks/routes for transmission of multidrug-resistant *Listeria* from these contaminated meats to buyers, consumers, and the public. VAN-resistant organisms are known to exhibit MDR [45]; interestingly, all the VAN-resistant strains in this study preferred antibacterial agents belonging to three or more classes. Moreover, MARI >0.2 indicates that an isolate originated from an environment with high antibacterial selection pressure [30]; thus, isolates in this study originated from high-risk sources of contamination since they all had MARI >0.2. This could also explain the cause of high MDR observed in this study.

## Conclusion

This study has shown that a sizeable proportion of raw meats (chicken meat 27.1%, pork 13.2%, and beef 45.8%) marketed in Enugu State Southeast Nigeria, is contaminated by diverse multidrug-resistant *Listeria* species. Thus, these meats are potential reservoirs and vehicles of transmission of multidrug-resistant *Listeria* and genes encoding resistance to many classes of antibacterial agents. The presence of these organisms in raw meats poses a danger to the health of handlers/consumers of these meats and the public in general. These individuals could serve as disseminators of these organisms into the environment. The spread of multidrug-resistant *Listeria* could have a huge impact on the ecology and epidemiology of ABR in Nigeria. Therefore, attention should be paid on the use of antibacterial agents in livestock production and in other settings since selection pressure in meat-yielding animals/environment could emanate from various sources. There is a need for continual monitoring of the emergence of resistant *Listeria* species in Nigeria because these organisms could transfer resistance genes to other clinically-relevant Gram-positive organisms. However, further studies to determine the genes encoding resistance to various antibacterial agents in the isolates are recommended.

## Authors’ Contributions

OJO conceptualized the study. EEU, MNI, COO, COA, ACM, and OLM collected and processed the samples. EVE and OJO analyzed the results. MUA and OJO drafted the manuscript while ROV and MV did the molecular characterization. All authors read and approved the final manuscript.
